# CD200 Modulates *S. aureus*-Induced Innate Immune Responses Through Suppressing p38 Signaling

**DOI:** 10.3390/ijms20030659

**Published:** 2019-02-03

**Authors:** Bo Zhu, Yingying Yu, Xiaoyi Liu, Qin Han, Yanhua Kang, Liyun Shi

**Affiliations:** 1 School of Medicine and Life Sciences, Nanjing University of Chinese Medicine, Nanjing 210023, China; zhuyingxiaoge@163.com (B.Z.); liuxiaoyi1993@outlook.com (X.L.); hanqin0606@163.com (Q.H.); 13758140215@163.com (Y.K.); 2 School of Medicine, Hangzhou Normal University, Hangzhou, Zhejiang 310036, China; 13675857856@139.com

**Keywords:** CD200, *Staphylococcus aureus*, macrophage, p38 MAPK

## Abstract

Rapid activation of macrophages plays a central role in eliminating invading bacteria as well as in triggering the inflammatory responses, but how the anti-bacterial and the inflammatory responses are coordinated, in terms of macrophages, is not completely understood. In this study, we demonstrated that *Staphylococcus aureus* (*S. aureus*) induced the expression of CD200 in murine macrophages in a dose-dependent manner. We found that CD200 significantly suppressed the *S. aureus*-induced production of nitric oxide and proinflammatory cytokines in mouse macrophages. Concurrently, the bactericidal capability of macrophages was boosted upon the deletion of CD200. Furthermore, our data demonstrated that p38 mitogen-activated protein kinase (MAPK) was selectively down-regulated by CD200 administration, while enhanced upon CD200 silence in response to staphylococcal infection. The negative effect of CD200 siRNA on NO production in macrophages was largely abrogated upon the inhibition of p38 signaling, implying its critical involvement in this regulation. Together, our data demonstrate that CD200 plays a central role in regulating the inflammatory responses and the anti-bacterial activity of macrophages, at least partially, through suppressing p38 activity.

## 1. Introduction

Methicillin-resistant *Staphylococcus aureus* (MRSA) has become a main cause of hospital-associated infections, with high morbidity and mortality rates [1]. As a central component of antimicrobial host defense, macrophages play a critical role in preventing and limiting MRSA infections by recognizing, engulfing, and eliminating the pathogens [2]. Proinflammatory cytokines produced and released by macrophages activate various immune cells and protect against *S. aureus* infection effectively. However, excessive inflammatory responses in macrophages evoked by *S. aureus* often cause tissue injury in the host [3]. Hence, the homeostasis of immune responses against *S. aureus* in macrophages should be tightly regulated. The molecular mechanisms underlying immune homeostasis in *S. aureus*-triggered macrophage activation are yet to be elucidated.

Numerous inhibitory molecules have been described on macrophages and are involved in the control of inflammatory responses [4]. The transmembrane protein CD200 is one of the best-characterized immune inhibitory molecules [5,6]. CD200 is widely expressed, whereas its receptor CD200R is expressed mainly on myeloid cells, including macrophages [7]. The CD200/CD200R immune-checkpoint pathway has been widely demonstrated to maintain immune homeostasis during infection by preventing excessive activation of macrophages [8,9,10,11]. However, these studies are mainly conducted in models of virus infection [8,9,10,11]. The effects and underlying mechanisms of CD200 on bacterial-triggered macrophage activation remain elusive.

In the present study, we explored the role and underlying mechanisms of CD200 in *S. aureus*-induced innate immune responses. Our study aims to provide more therapeutic opportunities for MRSA infection based on the immune checkpoint molecule CD200.

## 2. Results

### 2.1. CD200 Expression Was Induced upon S. aureus Infection in Murine Macrophages

**Figure 1:**
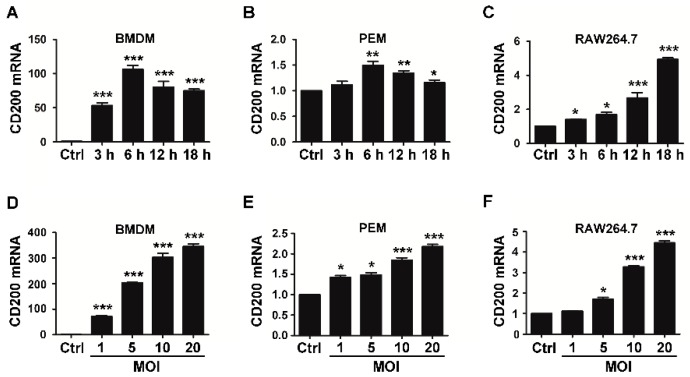
*S. aureus* infection induces CD200 expression in murine macrophages. Mouse BMDMs (**A**,**D**), PEMs (**B**,**E**), or RAW264.7 cells (**C**,**F**) were challenged with *S. aureus* (MOI = 1) for the indicated time periods (0–18 h), or with *S. aureus* at the indicated MOIs (0–20) for 6 h. Cells were then collected and detected for CD200 mRNA level by qPCR. Results are expressed as the mean ± SD of three independent experiments; * *p* < 0.05, ** *p* < 0.01, *** *p* < 0.005 versus Ctrl.

To explore the role of CD200 in macrophage responses to *S. aureus*, we first detected the expression pattern of CD200 during bacterial infection. Mouse bone marrow-derived macrophages (BMDMs), peritoneal macrophages (PEMs), or RAW264.7 macrophages were stimulated with *S. aureus* (MOI = 1) for the indicated time periods. We found that CD200 mRNA level was increased in all these macrophages upon the stimulation of *S. aureus* (Figure 1A–C). To further verify the induction of CD200 by Staphylococcal infection, mouse BMDMs, PEMs, or RAW264.7 macrophages were challenged with various amounts of *S. aureus* (MOI = 1–20) for 6 h. The result showed that Staphylococcal infection induced the expression of CD200 in a dose-dependent manner (Figure 1D–F).

### 2.2. CD200 Inhibits Inflammatory Cytokines Production Triggered by S. aureus in Mouse Macrophages

**Figure 2:**
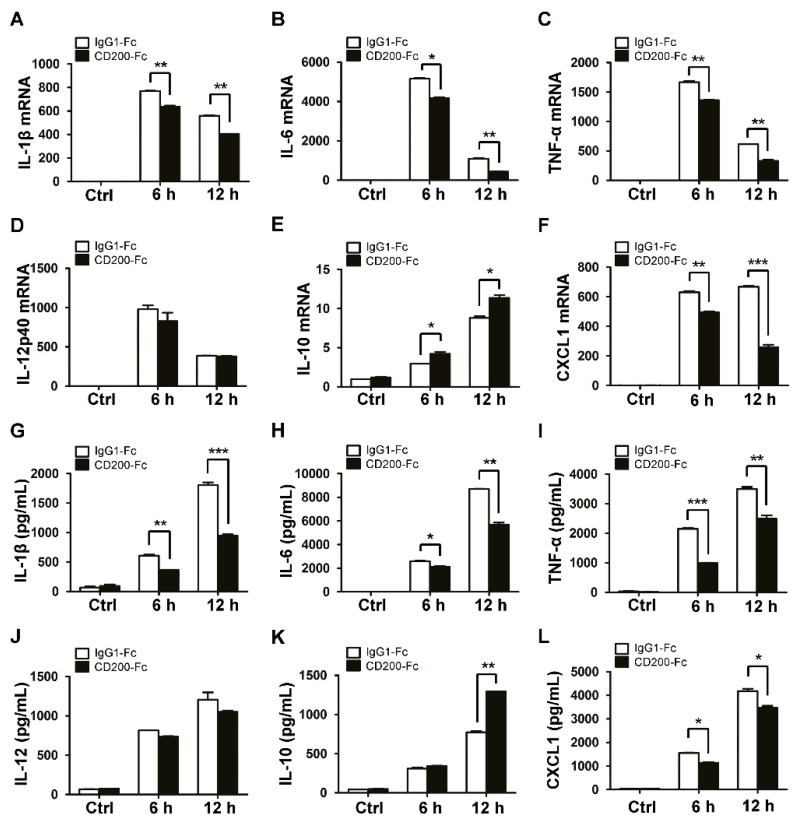
CD200 inhibits the production of inflammatory cytokines induced by *S. aureus*. Mouse BMDMs were pre-treated with CD200-Fc (2 μg/mL) or IgG1-Fc (2 μg/mL) for 1 h, and then stimulated with *S. aureus* (MOI = 1) for indicated time periods (0–12 h). (**A**–**F**) Relative mRNA expression levels of IL-1β (**A**), IL-6 (**B**), TNF-α (**C**), IL-12p40 (**D**), IL-10 (**E**), or CXCL1 (**F**) were detected by qPCR, with β-actin as an internal control. (**G**–**L**) The amount of IL-1β (**G**), IL-6 (**H**), TNF-α (**I**), IL-12 (**J**), IL-10 (**K**), or CXCL1 (**L**) in the cell culture supernatant was determined by ELISA. Results are expressed as the mean ± SD of three independent experiments; * *p* < 0.05, ** *p* < 0.01, *** *p* < 0.005.

**Figure 3:**
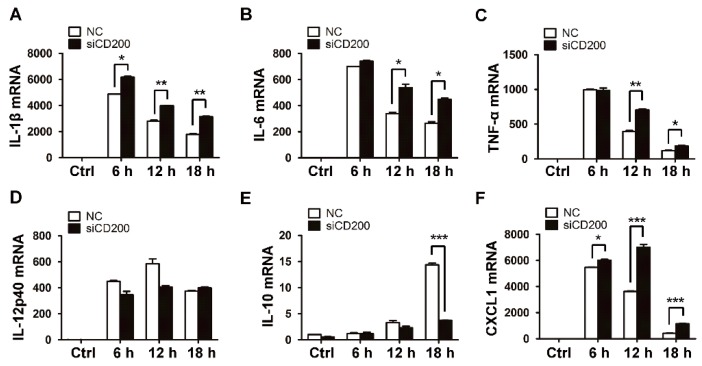

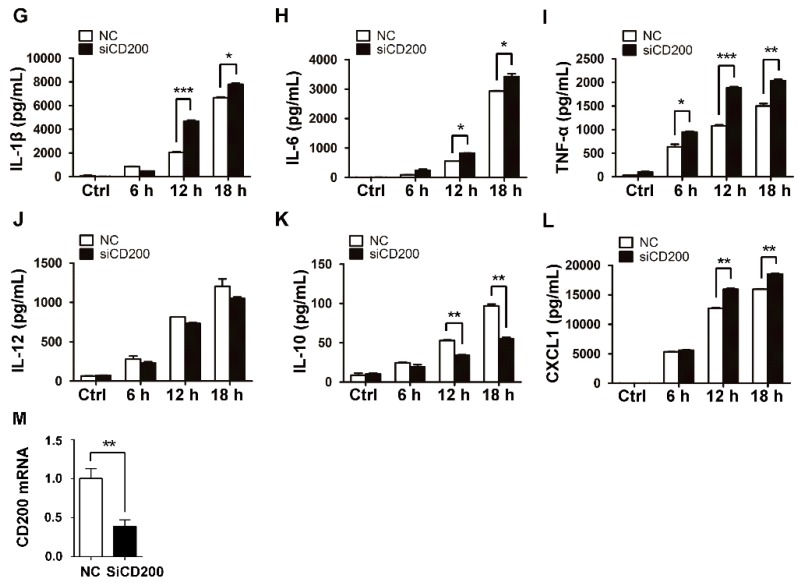
Knockdown of CD200 enhances *S. aureus*-induced inflammatory responses. Mouse PEMs were transfected with siCD200 or NC siRNA for 48 h, and then stimulated with *S. aureus* (MOI = 1) for indicated time periods (0–18 h). (**A**–**F**) Relative mRNA levels of IL-1β (**A**), IL-6 (**B**), TNF-α (**C**), IL-12p40 (**D**), IL-10 (**E**), or CXCL1 (**F**) were detected by qPCR, with β-actin as an internal control. (**G**–**L**) The amount of IL-1β (**G**), IL-6 (**H**), TNF-α (**I**), IL-12 (**J**), IL-10 (**K**), or CXCL1 (**L**) in the cell culture supernatant was determined by ELISA. (**M**) The CD200 knockdown efficiency was detected by qPCR. Results are expressed as the mean ± SD of three independent experiments; * *p* < 0.05, ** *p* < 0.01, *** *p* < 0.005.

Since the inflammatory response is primarily triggered upon bacterial infection, we next explored the potential role of CD200 in regulating the production of inflammatory cytokines by *S. aureus*-infected macrophages. To this end, mouse BMDMs were pre-treated with CD200-Fc (a solubilized form of CD200 that is often used as the agonist of CD200R1) or the control protein, IgG1-Fc, for 1 h, and then challenged with *S. aureus* (MOI = 1) for indicated time periods. The mRNA and protein levels of the inflammatory cytokines were evaluated by qPCR and ELISA, respectively. Strikingly, CD200-Fc, but not IgG1-Fc, significantly inhibited the expression of proinflammatory cytokines, including IL-1β, IL-6, TNF-α, IL-12, or CXCL1, both at mRNA (Figure 2A–D,F) and protein (Figure 2G–J,L) levels. Conversely, the expression of the anti-inflammatory cytokine IL-10 was found to be boosted upon CD200-Fc treatment (Figure 2E,K). To further substantiate the effect of CD200 on *S. aureus*-triggered inflammatory responses, we then transfected mouse PEMs with CD200-targeted or non-specific (NC) siRNA, followed by Staphylococcal infection. The results showed that the interference of CD200 expression in macrophages significantly enhanced the production of proinflammatory cytokines but inhibited the expression of anti-inflammatory cytokine IL-10 (Figure 3).

### 2.3. CD200 Affects Polarization and Compromises Bactericidal Activity of Macrophages

**Figure 4:**
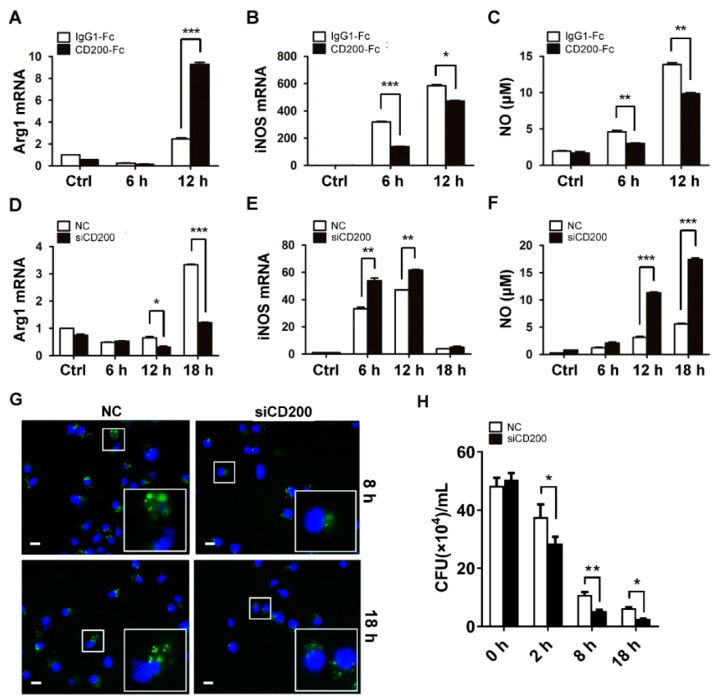
CD200 signaling inhibits NO synthesis and bactericidal activity of *S. aureus*-infected macrophages. (**A**–**C**) Mouse BMDMs were pre-treated with CD200-Fc (2 μg/mL) or IgG1-Fc (2 μg/mL) for 1 h, and then stimulated with *S. aureus* (MOI = 1) for indicated time periods (0–12 h). Relative mRNA levels of Arg1 (**A**) or iNOS (**B**) were detected by qPCR. NO release was determined using Griess reagent system (**C**). (**D**–**F**) Mouse PEMs were transfected with siCD200 or NC siRNA for 48 h, and then stimulated with *S. aureus* (MOI = 1) for indicated time periods (0–18 h). Arg1 (**D**) and iNOS (**E**) mRNA levels and NO release (**F**) were determined. (**G**) PEMs transfected with siCD200 or NC siRNA were challenged with CFSE-labeled *S. aureus* (MOI = 10) for indicated time periods (0–18 h). Cells were collected, fixed, and stained with DAPI. Intracellular bacterial were observed using a fluorescence microscope. A partially enlarged view was shown in the lower left corner. Scale bars: 10 µm. Shown are representative images from three independent experiments. (**H**) Mouse PEMs were transfected with siCD200 or NC siRNA for 48 h, and then challenged with *S. aureus* (MOI = 10) for indicated time periods (0–18 h). Cells were lysed and the intracellular bacterial burden was determined by CFU counting. Results are expressed as the mean ± SD of three independent experiments; * *p* < 0.05, ** *p* < 0.01, *** *p* < 0.005.

Macrophage polarization has been demonstrated to be essential in determining the outcome of infectious diseases [12,13]. The proinflammatory M1 subtypes mainly process the bactericidal potential and promote pathogen clearance, whereas the M2 subtypes exert the immunomodulatory effect and contribute to tissue repair [14]. The inhibitory effects of CD200 on the production of proinflammatory cytokines suggested that it might promote an M1- to M2-phenotype transition during bacterial infection. Using CD200-Fc or CD200 siRNA, we found that the engagement of CD200R remarkably enhanced the expression of the M2 marker Arg1 (Figure 4A,D), while inhibiting the expression of the M1 featured molecule iNOS (Figure 4B,E). Congruent with this, the release of NO triggered by *S. aureus* was reduced by CD200-Fc treatment but boosted upon CD200 knockdown (Figure 4C,F).

Given the importance of macrophages in innate immunity against bacterial infection, we then analyzed the effect of CD200 on macrophage bactericidal activity. To this end, murine macrophages were transfected with CD200 siRNA or NC, followed by the infection of CFSE-labeled *S. aureus* for 8 or 18 h. Cells were then collected and the amounts of the intracellular bacteria were observed under a fluorescence microscope. We also compared the intracellular bacterial burden in macrophages transfected with CD200 siRNA or control siRNA by CFU counting. Notably, the loads of intracellular bacteria were significantly reduced in CD200–silenced macrophages, as compared with that in control cells (Figure 4G,H). The results thus indicated that CD200 exerted an inhibitory effect on macrophage bactericidal activity.

### 2.4. CD200 Mildly Attenuates NF-κB Activation in Response to S. aureus Infection

**Figure 5:**
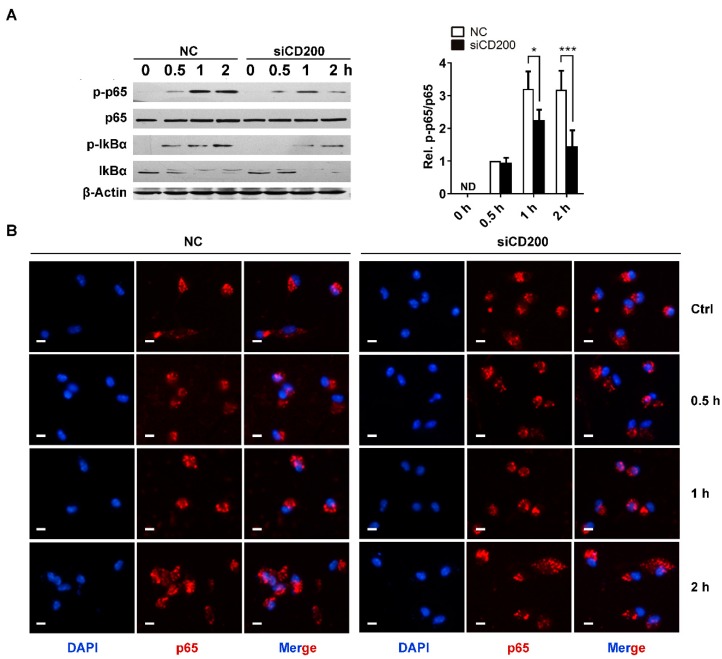
CD200 mildly affects NF-κB activation triggered by Staphylococcal infection. Mouse PEMs were transfected with CD200 siRNA or NC siRNA for 48 h, and then stimulated with *S. aureus* (MOI = 1) for indicated time periods (0–2 h). (**A**) Cells were lysed, and the protein level of p-p65, p65, p-IκBα, or IκBα was detected by western blotting. β-Actin served as a loading control. (**B**) Immunofluorescence was performed to visualize p65 nuclear translocation. Scale bars: 10 µm. Representative images of three independent experiments are shown. The phosphorylation state of p65 was semi-quantitated using ImageJ software. ND means not detected; * *p* < 0.05, *** *p* < 0.005.

NF-κB pathway has been demonstrated to play a key role in regulating macrophage activation and polarization [15,16,17,18]. To explore the mechanism underlying the immunosuppressive effects of CD200 on macrophages, we detected the effects of CD200 siRNA on NF-κB activation in response to Staphylococcal infection. Unexpectedly, we found that phosphorylation of p65 triggered by *S. aureus* was attenuated upon CD200 knockdown in mouse PEMs (Figure 5A). The nuclear translocation of NF-κB p65 was not affected by CD200 knockdown (Figure 5B). These data indicated that CD200 knockdown enhanced *S. aureus*-induced macrophage activity through other mechanisms rather than through promoting NF-κB activation.

### 2.5. CD200 Inhibits p38 Activation Triggered by S. aureus Stimulation

**Figure 6:**
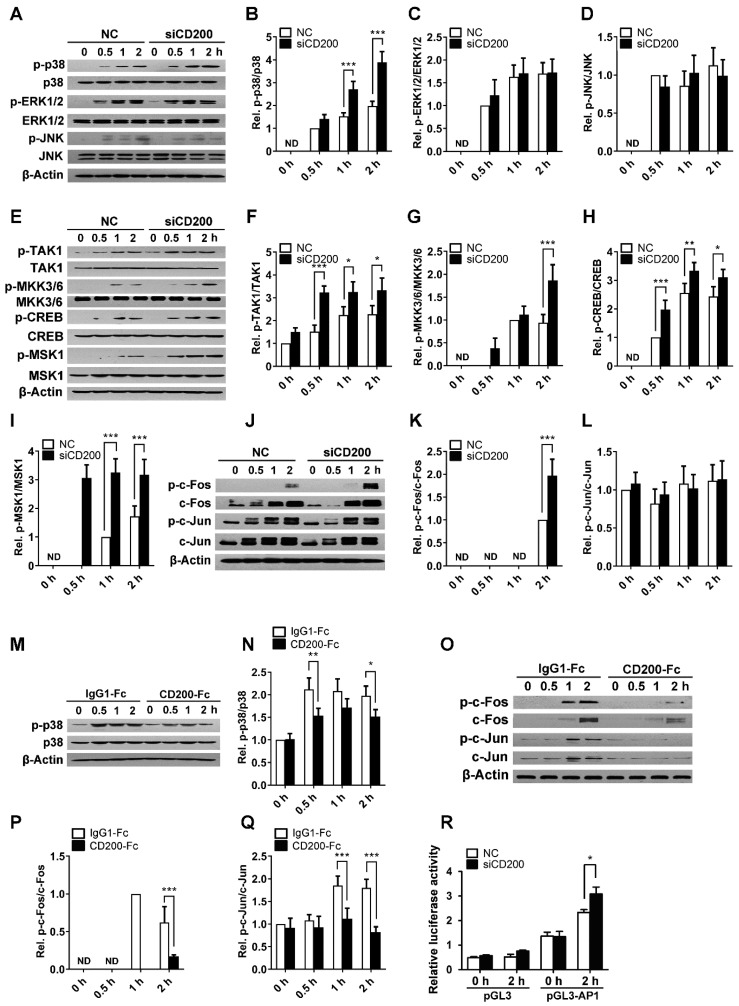
CD200 restrains p38 signaling upon Staphylococcal infection. (**A**–**L**) Mouse PEMs were transfected with CD200 siRNA (siCD200) or control siRNA (NC) for 48 h, and then stimulated with *S. aureus* (MOI = 1) for indicated time periods. The protein expression levels of p-p38, p38, p-ERK1/2, ERK1/2, p-JNK, JNK, p-TAK1, TAK1, p-MMK3/6, MMK3/6, p-CREB, CREB, p-MSK1, MSK, p-c-Fos, c-Fos, p-c-Jun, and c-Jun were detected by western blotting. (**M**–**Q**) Mouse BMDMs were pre-treated with CD200-Fc (2 μg/mL) or IgG1-Fc (2 μg/mL) for 1 h, and then stimulated with *S. aureus* (MOI = 1) for indicated time periods. The protein expression levels of p-p38, p38, p-c-Fos, c-Fos, p-c-Jun, and c-Jun were detected by western blotting. (**R**) RAW264.7 macrophages were pre-transfected with CD200 siRNA for 48 h and then challenged with *S. aureus* (MOI = 10) for 2 h. The AP-1 luciferase activity was analyzed by the dual luciferase. Representative blots from three independent experiments are shown (**A**,**E**,**J**,**M**, and **O**). Changes in the phosphorylation state of different proteins were semi-quantitated using ImageJ software (**B**–**D**,**F**–**I**,**K**–**L**,**N**,**P**–**Q**). ND means not detected; * *p* < 0.05, ** *p* < 0.01, *** *p* < 0.005.

Mitogen-activated protein kinases (MAPKs), including the p38 MAPK, the extracellular-regulated kinase 1/2 (ERK1/2), and the c-Jun N-terminal kinase (JNK), play a key role in immune and inflammatory responses [19,20,21,22,23]. In the present study, we found that *S. aureus* stimulation activated three major MAPKs in mouse PEMs, among which, the activation of p38 MAPK was significantly enhanced upon CD200 siRNA treatment (Figure 6A–D). In line with this, the phosphorylation of p38 triggered by *S. aureus* was blunted upon the engagement of CD200R (Figure 6M,N), indicating the negative regulation of p38 by CD200. Supportively, the activity of the upstream kinases including MAPK kinase 3/6 (MKK3/6) and TGF-β activated kinase 1 (TAK1), or the activation of the downstream MSK1/CREB pathway, was consistently enhanced by CD200 siRNA in response to *S. aureus* stimulation (Figure 6E–I). The activation of AP-1, including c-Fos and c-Jun, is controlled by p38 MAPK pathway. We found that the phosphorylation of c-Fos triggered by *S. aureus* infection was significantly enhanced by CD200 knockdown (Figure 6J–L). We also found that the phosphorylation of c-Fos and that of c-Jun was suppressed by CD200-Fc treatment in macrophages during *S. aureus* infection (Figure 6O–Q). Results from the dual-luciferase reporter assay further confirmed the down-regulatory effects of CD200 on AP-1 activity (Figure 6R). These data thus supported that p38 MAPK was critically involved in CD200-mediated regulation of macrophage activity during bacterial infection.

### 2.6. CD200 Blunts NO Synthesis Through Regulating p38 Activity

**Figure 7:**
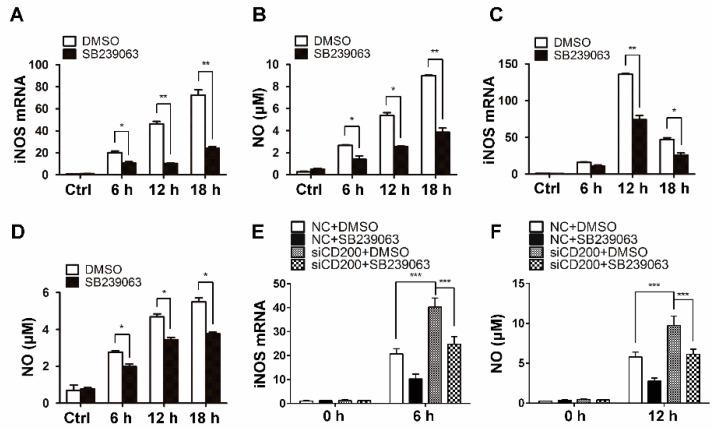
The p38 inhibition largely abolished the effects of CD200 on NO synthesis induced by staphylococcal infection. (**A**–**D**) Mouse PEMs (**A**,**B**) or BMDMs (**C**,**D**) were pre-treated with SB239063 (10 μM, 0.5 h) or vehicle (DMSO), and then treated with *S. aureus* for indicated time periods (0–18 h). The mRNA level of iNOS was detected by qPCR, and the concentration of NO in culture supernatants was determined using Griess reagent. (**E**,**F**) Mouse PEMs were transfected with CD200 siRNA or NC for 48 h and treated with SB239063 or vehicle (DMSO) for 0.5 h. Cells were then infected with *S. aureus* for indicated time periods (0–12 h). The mRNA level of iNOS and the concentration of NO in culture supernatants were analyzed. Results are expressed as the mean ± SD of three independent experiments; * *p* < 0.05, ** *p* < 0.01, *** *p* < 0.005.

We next sought to understand the functional relevance of p38 MAPK with macrophage and CD200 regulation. Considering that NO is a featured proinflammatory cytokine essential for macrophage bactericidal activity, we thus assessed the effect of p38 inhibition on NO production by *S. aureus*-stimulated macrophages. Indeed, our data showed that the treatment of p38 inhibitor SB239063 significantly inhibited iNOS mRNA expression and NO release in both mouse PEMs (Figure 7A,B) and BMDMs (Figure 7C,D) upon Staphylococcal infection. Moreover, the enhanced NO synthesis induced by CD200-silenced macrophages was notably resumed upon SB239063 treatment (Figure 7E,F). The data suggested that the negative regulation of macrophage activity by CD200 was largely dependent on its modulation of p38 MAPK pathway.

## 3. Discussion

Inflammation needs to be tightly controlled to avoid immunopathology. The mechanism maintaining immune homeostasis during *S. aureus* infection is not completely understood. In the present study, we demonstrated that CD200 inhibited proinflammatory cytokine production, NO synthesis, and bactericidal activity in macrophages infected with *S. aureus*, at least partially, through downregulating p38 activity. Our results indicate that CD200 plays a key role in maintaining innate immune homeostasis against *S. aureus* infection.

The immune inhibitory effects of the CD200/CD200R1 signaling pathway have been widely reported in myeloid cells, including mast cells [24], dendritic cells [25], basophils [26], and macrophages [27]. Mice lacking CD200 have increased amounts of myeloid cells, and are more susceptible to experimental autoimmune disorders [28]. Hoek et al. has demonstrated that CD200 downregulates macrophage lineage cells, including brain microglia [27]. Although the suppressive role of CD200 in antiviral immunity has been previously investigated [8,9,11], the function of CD200 in anti-bacterial response remains elusive. CD200 induction by TLRs and NOD-like receptors has been demonstrated to limit macrophage activation and to protect the host from *Meningococcal Septicemia* [29]. Consistently, we found that *S. aureus* stimulation significantly upregulated CD200 expression in mouse macrophages. Increased CD200 expression during *S. aureus* infection indicates its importance in anti-bacterial immunity. Importantly, we demonstrated that CD200 suppressed inflammatory cytokine production and NO release in *S. aureus*-challenged macrophages. The changes in NO synthesis indicated a potential regulatory effect of CD200 on macrophage polarization during *S. aureus* infection. Consistently, the expression of the inhibitory CD200R has been described as a potential marker of the alternative macrophage activation [30]. The mechanism underlying the regulatory effects of CD200/CD200R axis on the expression of iNOS and downstream macrophage phenotype transition deserves further investigation.

NF-κB is a critical transcription factor that regulates innate immune response [16]. The activation of NF-κB pathway has been widely accepted as an essential mechanism for proinflammatory cytokine production in different immune cells, including macrophages. Recently, Jiang et al. demonstrated that CD200 reduces TLR4-mediated inflammatory responses in LPS-treated rat primary microglial cells through inhibiting the NF-κB pathway [31]. CD200 has also been described to suppress NF-κB activity in IFN-γ/LPS-stimulated rat macrophages [32]. In contrast, we found that the knock-down of CD200 even mildly attenuated NF-κB activation in mouse PEMs stimulated with *S. aureus*. LPS is the main endoxin of Gram-negative bacterial, while *S. aureus* is a typical representative of Gram-positive bacterial. The bidirectional and complex effects of CD200 on NF-κB pathway may depend on different pathogen types.

Various pathogens or PRRs have been reported to trigger immune responses through activating MAPK pathways [19,33]. CD200R engagement inhibits cytokine production in mast cells via inhibiting MAPKs, including p38 MAPK, ERK1/2, and JNK pathway [24]. Here, we demonstrated that CD200 inhibited the activation of p38 MAPK, but not ERK1/2 or JNK pathways, induced by *S. aureus* stimulation in mouse macrophages. We further demonstrated that the inhibitory effects of CD200 siRNA on *S. aureus*-stimulated-NO synthesis could be reversed by p38 inhibitor pre-treatment. These data indicate that CD200/p38 axis plays an important role in controlling innate immune homeostasis during *S. aureus* infection.

Both the p38/MSK-1/CREB axis and the p38/AP-1 axis are involved in immune regulation in myeloid cells [34,35,36]. The p38/MSK-1/CREB axis influences cytokine production in human neutrophils [34]. As recently reported, PSM peptides of *S. aureus* modulate cytokine production via activating the p38/MSK-1/CREB axis in dendritic cells [35]. Similarly, the p38/AP-1 pathway regulates LPS-induced inflammatory responses in macrophages [36]. In the present study, we found that CD200 down-regulated both the p38/MSK-1/CREB pathway and the p38/AP-1 pathway in macrophages during *S. aureus* infection. These results highlighted the importance of p38 MAPK in immune balance as a downstream pathway of CD200 in macrophages infected with *S. aureus*. The exact role of the p38/MSK-1/CREB axis and p38/AP-1 axis in regulating the immunosuppressive effects of CD200 on innate immune responses remains unclear and deserves further investigation.

In conclusion, we identified CD200 as an inflammatory suppressor in macrophages infected with *S. aureus*. The CD200/p38 signal axis plays an important role in controlling innate immune responses against *S. aureus* infection.

## 4. Materials and Methods

### 4.1. Mice

C57BL/6 female mice (6–8 week-old) were obtained from the Shanghai Experimental Animal Center of Chinese Academy of Sciences (Shanghai, China). All animal experiments were performed in accordance with the National Institutes of Health Guide for the Care and Use of Laboratory Animals, and approved by the Animal Care and Use Committee of Nanjing University of Chinese Medicine (ACU-57, 30 December 2016).

### 4.2. Reagents

Thioglycollate, 4′,6-diamidine-2-phenylindole dihydrochloride (DAPI), SB239063 were purchased from Sigma-Aldrich (St. Louis, MO, USA). M-CSF, recombinant mouse CD200 Fc chimera, or IgG Fc chimera were purchased from R&D Systems (Minneapolis, MN, USA). The following antibodies were purchased from Cell Signaling Technology (Danvers, MA, USA): p65, p-p65, IκBα, p-IκBα, p38, p-p38, ERK1/2, p-ERK1/2, JNK, p-JNK, TAK1, p-TAK1, MKK3/6, p-MKK3/6, CREB, p-CREB, MSK1, p-MSK1, c-Fos, p-c-Fos, c-Jun, p-c-Jun, and β-actin.

### 4.3. Preparation of S. aureus

One day before the experiment, the MRSA strain USA300 was grown to the stationary growth phase overnight at 37 °C in Luria-Bertani broth with agitation (180 rpm). On the next morning, the bacteria were diluted (OD_600_ around 0.1) and cultured for another two hours until the OD_600_ reach 0.6 to 0.8. The bacteria were harvested by centrifugation (4000× *g*, 5 min), washed with, and re-suspended, in PBS. The bacterial densities were estimated at OD_600_. OD_600_ of 1.0 is approximately 1.5 × 10^9^ CFU/mL.

### 4.4. Cell Culture

Mouse BMDMs were isolated as previously described [37]. Briefly, bone marrow cells were isolated by flushing tibias and femurs of mice. BMDMs were differentiated in DMEM supplemented with 10% heat-inactivated fetal bovine serum (FBS), 15% filtered L929 cell culture supernatant, 10 ng/mL M-CSF, 100 units/mL penicillin, and 100 μg/mL streptomycin. The cells were changed with fresh medium every 3 days, and then harvested on day 7 for further analysis. To prepare PEMs, 6 week-old mice were injected intraperitoneally with 2 mL of 3% thioglycollate. After 4 days, total peritoneal exudate cells were harvested by peritoneal lavage with PBS. The cells were cultured in DMEM supplemented with 10% heat-inactivated FBS, 100 units/mL penicillin, and 100 μg/mL streptomycin. After culturing for 2 h, non-adherent cells were removed by gently washing with PBS, and the attached macrophages were maintained for subsequent experiments. The macrophage-like cell line RAW264.7 was cultured in DMEM supplemented with 10% heat-inactivated FBS, 100 units/mL penicillin, and 100 μg/mL streptomycin. All cells were maintained in a humidified 5% CO2 atmosphere at 37 °C.

### 4.5. Transfection of siRNA

The siRNA molecules targeting CD200 mRNA were purchased from GenePharma Corporation (Shanghai, China), and delivered into macrophages using X-tremeGENE siRNA transfection reagent (Roche, Basel, Switzerland), according to the manufacturer’s instructions.

### 4.6. RNA Isolation, Reverse Transcription, and qRT-PCR

Total RNA was isolated from BMDMs, PEMs, or RAW264.7 cells with the TRIzol reagent (Thermo Fisher Scientific, Waltham, MA, USA) according to the manufacturer’s instructions. First-strand cDNA was synthesized from 1 μg of RNA using the PrimerScript II 1st Stand cDNA Synthesis Kit (Takara, Tokyo, Japan). Quantitative real-time PCR was performed on an ABI 7500 real-time PCR system using SYBR Green PCR Master Mix (Thermo Scientific, MA, USA). The following primers were used: CD200 forward, 5′-AGTGGTGACCCAGGATGAA-3′ and reverse, 5′-TACTATGGGCTGTACATAG-3′; IL-6 forward, 5′-CCACTTCACAAGTCGGAGGCTTA-3′ and reverse, 5′-AGTGCATCATCGTTGTTCATAC-3′; iNOS forward, 5′-CCCTTCCGAAGTTTCTGGCAGCAGCG-3′ and reverse, 5′-GGCTGTCAGAGCCTCGTGGCTTTGG-3′; TNF-α forward, 5′-AAGGCCGGGGTGTCCTGGAG -3′ and reverse, 5′-AGGCCAGGTGGGGACAGCTC-3′; IL-1β forward, 5′-GAAATGCCACCTTTTGACAGTG-3′ and reverse 5′- TGGATGCTCTCATCAGGACAG-3′; IL-10 forward, 5′- CTTACTGACTGGCATGAGGATCA-3′ and reverse 5′-GCAGCTCTAGGAGCATGTGG-3′; IL-12 p40 forward, 5′-TCATCAGGGACATCATCAAAC-3′ and reverse 5′- TGAGGGAGAAGTAGGAATGGG-3′; CXCL1 forward, 5′- ACTCAAGAATGGTCGCGAGG-3′ and reverse 5′- GTGCCATCAGAGCAGTCTGT-3′; Arg1 forward, 5′-TGAACACGGCAGTGGCTTTA-3′ and reverse 5′-GCATTCACAGTCACTTAGGTGGTTTA-3′; β-actin forward, 5′-CTCATGAAGATCCTGACCGAG-3′, and reverse, 5′- AGTCTAGAGCAACATAGCACAG-3′. The relative mRNA expression level of target genes was calculated by the 2^−ΔΔCt^ method, with β-actin serving as an internal reference.

### 4.7. Cytokine Measurements by ELISA

IL-6, TNF-α, CXCL1, IL-1β, IL-10, or IL-12 in culture supernatants were measured using commercially available ELISA kits (R&D Systems, Minneapolis, MN, USA), according to the guidelines of the manufacture.

### 4.8. Nitric Oxide (NO) Measurement

*S. aureus*-induced NO production in culture supernatants was measured using Griess reagent system by ELISA method (Promega, Madison, USA), according to the manufacturer’s instructions.

### 4.9. Bacterial Killing Assay

As previously described [38], the bactericidal capability of macrophages was observed using a fluorescence microscope or determined by CFU counting. *S. aureus* were collected by centrifugation (4000× *g*, 5 min), washed twice with PBS, and re-suspended in PBS to an OD_600_ of 1.0. The bacterial suspension was incubated at 37°C for 15 min in the dark with an equal volume of PBS containing 4.0 μM 5-(and-6-)-carboxyfluorescein diacetate, succinimidyl ester (CFDA-SE). The labeling reaction was stopped by the addition of a 5-fold volume of ice-cold PBS. The CFSE-labeled bacteria were washed with PBS three times to remove excess dye, and re-suspended again in PBS for use. PEMs were grown on cover slips and incubated with CFSE-labeled S. aureus (MOI = 10) for indicated time periods (0–18 h). The infected PEMs were collected, fixed, and stained with DAPI. Intracellular bacterial load were observed using a fluorescence microscope. PEMs stimulated with S. aureus (MOI = 10) for the indicated time periods (0–18 h) were washed with PBS three times, treated with gentamycin (200 μg/mL, to kill extracellular bacterial), and lysed with 200 μL 1% Triton-100. Intracellular bacterial burden was determined by plating serial dilutions in LB and incubating at 37 °C overnight. Count of bacterial CFU was performed on plates.

### 4.10. Western blotting

Murine PEMs or BMDMs were harvested and lysed in ice-precooled RIPA lysis buffer. Lysates were then centrifuged at 12,000× *g* for 10 min at 4 °C, and the clear supernatants were obtained. Protein concentrations were quantified by BCA assay (Beyotime Biotechnology, Nantong, China). Proteins were separated by 10% SDS-PAGE and transferred to PVDF membrane by wet transfer at 300 mA for 1.5 h. After being blocked with 5% non-fat milk in TBS-Tween buffer (TBST, containing 20 mM Tris, 500 mM NaCl and 0.1% Tween 20) for 1 h at room temperature, the membranes were probed with indicated primary antibodies overnight at 4 °C. After washing three times with TBST, the membranes were incubated with horseradish-peroxidase conjugated secondary anti-rabbit or anti-mouse antibody for 1 h at room temperature. The blots were washed for another round and the protein was detected using enhanced chemiluminescence (Cell Signaling Technology, Danvers, MA, USA). The semi-quantitative analysis was performed using the ImageJ software (NIH, Bethesda, MD, USA).

### 4.11. Immunofluorescence

PEMs were plated on coverslips, transfected with CD200 siRNA for 48 h, and then stimulated with *S. aureus* (MOI = 1) for indicated time periods (0–2 h). PEMs were fixed with 100% methanol for 10 min at −20 °C, washed three times with PBS, and then permeabilized with 0.2% saponin for 10 min at room temperature. After another round of washes, cells were blocked with 5% BSA for 45 min at room temperature, and then incubated with anti-p65 antibody (1:200) in PBS containing 2.5% BSA at 4 °C overnight. After washing with PBS three times, cells were incubated with the secondary antibody conjugated with Texas Red in PBS containing 2.5% BSA for 1 h and rinsed in PBS again. The nuclei of cells were stained with DAPI (1 μg/mL) for 3 min. Images were obtained under fluorescence microscope.

### 4.12. Dual-Luciferase Assay

The Ap-1 activity was detected by Dual-Luciferase Reporter Assay. RAW264.7 cells were plated onto 24-well plates and transfected with CD200 siRNA or NC siRNA. After 24 h, cells were co-transfected with pRL-CMV-Luc and pGL3-AP-1 (or pGL3 basic vector) for another 48 h. Cells were stimulated with *S. aureus* (MOI = 10) or not, harvested, and lysed 2 h after stimulation. Luciferase activity was measured using the Dual-Luciferase Reporter Assay System, according to the manufacturer’s instructions (Promega, Madison, USA). Firefly luciferase activity was normalized to Renilla luciferase activity.

### 4.13. Statistical Analysis

Data are expressed as mean ± SD. Student’s t test, one-way ANOVA, or two-way ANOVA, followed by a multiple comparison (Bonferroni’s post-hoc test), were performed on a GraphPad Prism software package (Version 6.02, La Jolla, CA, USA). Significant differences were accepted when *p* < 0.05.

## Abbreviations

MRSA	Methicillin-resistant *Staphylococcus aureus*
*S. aureus*	*Staphylococcus aureus*
DAPI	4′,6-diamidine-2-phenylindole dihydrochloride
FBS	Fetal bovine serum
BMDM	Bone marrow-derived macrophage
PEM	Peritoneal macrophage
MOI	Multiplicity of infection
CFU	Colony-forming units
ND	Not detected
ELISA	Enzyme-linked immunosorbent assay
IL	Interleukin
TNF	Tumor necrosis factor
NO	Nitric oxide
MAPK	Mitogen-activated protein kinase
ERK1/2	Extracellular signal-regulated kinase 1/2
JNK	c-Jun NH2-terminal kinase (JNK)
TAK1	Transforming growth factor-beta-activated kinase-1
MKK3/6	Mitogen-activated Protein Kinase 3/6
CREB	cAMP-regulated enhancer B
MSK1	Mitogen- and stress-activated protein kinase-1
